# Bumblebee visitation and pollen dynamics in *Palicourea coriacea* (Rubiaceae): does coflowering with congeneric species matter?

**DOI:** 10.1093/aobpla/plaf014

**Published:** 2025-03-13

**Authors:** Rocío Pérez-Barrales, Túlio Sá, Raphael Matias, Marco Túlio Furtado, Ebenezer Rodríguez, Julio Rabadán González, Hélder Consolaro, Cibele Cardoso Castro

**Affiliations:** Botany Department, Faculty of Science, University of Granada, Avenida de Fuente Nueva s/n, 18071 Granada, Spain; Graduate Program in Biodiversity, Federal Rural University of Pernambuco, Rua Dom Manoel de Medeiros, s/n, Recife 52171-900, Pernambuco, Brazil; Institute of Biosciences, Federal University of Jataí, BR 364, KM 195, 3800, Jataí 75804-068, Brazil; Institute of Biosciences, Federal University of Jataí, BR 364, KM 195, 3800, Jataí 75804-068, Brazil; Postgraduate Program in Ecology, Conservation and Biodiversity, Institute of Biology, Federal University of Uberlandia, Avenida Amazonas, 20, Uberlandia 38405-302, Brazil; Observation.org, Calle Gordal 8, 41940 Tomares, Sevilla, Spain; Postgraduate Program in Ecology, Conservation and Biodiversity, Institute of Biology, Federal University of Uberlandia, Avenida Amazonas, 20, Uberlandia 38405-302, Brazil; Biotechnology Institute, Federal University of Catalaõ, Avenida Dr. Lamartine Pinto de Avelar, 1120, Catalão 75704-020, Brazil; Graduate Program in Biodiversity, Federal Rural University of Pernambuco, Rua Dom Manoel de Medeiros, s/n, Recife 52171-900, Pernambuco, Brazil

**Keywords:** adaptive inaccuracy, distyly, pollination competition, pollen delivery, pollen deposition, reproductive interference

## Abstract

The flowering phenology of many closely related species in the Brazilian Cerrado coincides with the onset of the rainy season, where sequential flowering often occurs with some overlap. Transitioning from solitary flowering to coflowering with congeneric species may alter the pollination environment, affecting pollen delivery and deposition patterns. Coflowering among conspecifics concurrently requires pollination niche differentiation to minimize reproductive costs. This study tested these concepts in the distylous *Palicourea coriacea* during two flowering periods: early in the season when it flowered alone, and later with conspecific *P. officinalis*, also distylous. Pollination syndromes were assessed by measuring corolla length, nectar volume and sugar concentration, and reproductive organ height. *Palicourea coriacea* shows yellow and shorter corollas with higher sugar concentration in the nectar, while *P. officinalis* presents yellow to orange longer corollas with more diluted nectar, aligning with bee and hummingbird pollination syndromes, respectively, as reported in the literature. However, the species exhibited significant overlap in stigma and anthers height. The main floral visitor in the two species during the study was *Bombus pauloensis.* Visitation increased through the season, particularly in conspecific patches of *P. coriacea*, resulting in higher pollen delivery. In contrast, pollen deposition was similar or higher in congeneric patches with *P. officinalis* during the coflowering period. Visits to *P. coriacea* were higher than in *P. officinalis*, suggesting a bumblebee preference for the former. The study highlights the complex interplay between flowering phenology, floral traits, and pollinator behaviour in shaping reproductive outcomes and potential niche differentiation. While differences in flowering and flower morphology may prevent potential costs of pollinator sharing, the risk of reproductive interference remains significant. Future research should focus on comprehensive pollination dynamics throughout the entire flowering season, measuring pollinator behaviour, pollen dynamics and plant fitness, to further elucidate the mechanisms driving floral evolution and niche differentiation in sympatric species.

## Teaser

This study investigates the pollination ecology of *Palicourea coriacea* in the Brazilian Cerrado to understand if flowering alongside *P. officinalis* modifies pollinator preference and pollen movement patterns. *Palicourea coriacea* has yellow flowers with sugary nectar, and blooms approximately a month earlier than *P. coriacea* , a species with reddish flowers and diluted nectar. *Bombus paulensis* visited *P. coriacea* through the flowering season, as well as *P. officinalis* in the coflowering period. However, bumblebees prefered *P. coriacea* in congeneric patches, resulting in higher pollen delivery compared to patches with only *P. coriacea* . In contrast, pollen deposition patters remained similar or higher in the patches with the two species. The findings highlight the complex interplay between flowering phenology, floral traits, and pollinator behaviour. While sequential flowering may reduce the costs of pollinator sharing, the potential for reproductive interference remains significant since the two species display similar stigma and anther height.

## Introduction

Pollination studies are central to investigating the mechanisms that allow closely related species to cooccur in communities (Elzinga *et al.* 2007; Sargent and Ackerly 2008). Studies of congeneric sympatric species have yielded valuable insights into the ecological foundations of pollination competition, the associated reproductive costs, and the evolutionary implications for the diversity of floral traits (Armbruster *et al.* 1994; Mitchell *et al.* 2009; Muchhala *et al.* 2010; Pauw 2013). How a species influences the pollination environment of a closely related coflowering species depends on whether pollinator sharing creates a positive or negative relationship with fitness, depicting scenarios ranging from the competition (Mitchell *et al.* 2009; Muchhala and Thomson 2012) to facilitation (Moeller 2004; Ghazoul 2006). Nevertheless, pollinator sharing does not always result in competition or facilitation, as evidenced by neutral outcomes (Armbruster and McGuire 1991; Mesquita-Neto *et al.* 2018).

The empirical evidence of pollinator-mediated interactions has evidenced a growing trend: a consistent tendency of pollinator sharing more extensively than expected by chance, leading to pollinator mediated plant to plan interactions affecting pollen dynamics and transfer among coflowering species (Arceo-Gómez and Ashman 2011; Ashman and Arceo-Gómez 2013; Ashman *et al.* 2020). Data from correlative and experimental studies on the reproductive costs of heterospecific pollen transfer (Bell *et al.* 2005; Moragues and Traveset 2005; Morales and Traveset 2008; Ashman *et al.* 2020; Pérez-Barrales and Armbruster 2023), coupled with the reliance of most angiosperms on pollinators for reproduction (Ollerton *et al.* 2011), suggests that competition for pollination services is a prevalent phenomenon among plant species in communities. Consequently, competition for pollination commonly results in the divergence of the use of resources (resource partitioning) and the pollination niche as a strategy to reduce the negative cost of pollinator sharing (i.e. lower visitation, pollen limitation, or heterospecific pollen transfer). For instance, competition could drive the differentiation of traits, enabling plant species to attract distinct pollinator guilds (Pauw 2013). This entails changes in attraction traits to recruit different groups of pollinators, for example, through the chemistry of fragrance (Salzmann *et al.* 2006), in flower traits to limit access to rewards, such as longer or narrower flower tubes, or flower handling (Castellanos *et al.* 2004; Muchhala 2007; Reynolds *et al.* 2009). In contrast, plants can share pollinators if they use the body of pollinators to partition pollen placement, also requiring differentiation in the architecture of reproductive organs (Armbruster *et al.* 1994; Muchhala and Potts 2007; Newman and Anderson 2020). This, in turn, requires high accuracy in pollen placement, subsequent contact with stigmas, and consistent flower handling to avoid pollen placement from different species on the same spot (Armbruster *et al.* 1994, 2014; Muchhala and Potts 2007).

Another important axis of variation reducing the costs of pollinator sharing arises from the temporal separation of flowering seasons, leading to distinct flowering peaks and dispersing phenology across time (Stiles 1975, 1977; Poole *et al.* 1979). In seasonal habitats, achieving complete temporal separation of flowering along a wide seasonal timescale is largely influenced by predictable climatic factors. The transition from vegetative growth to flowering signifies an adjustment of phenological events to align with optimal growing conditions to enhance plant reproduction and fitness. This transition is often initiated by regional climatic conditions, such as the onset of the rainy season (Batalha and Martins 2004; Jones and Daehler 2018; Santos de Oliveira *et al.* 2021; Ferreira *et al.* 2024), daylength perception (Warner and Erwin 2003; Kim *et al.* 2022) or gradients in climate as determined by latitude (Landoni *et al.* 2024). Consequently, sympatric species may perceive these seasonal cues and exhibit different flowering peaks within the same season (Mesquita-Neto *et al.* 2015). By doing so, species can partition flowering time using different flowering peaks within the season, thereby reducing the likelihood of interspecific pollen transfer while still sharing pollinators (Stiles 1975; Boas *et al.* 2013). When species experience substantial overlap in flowering along the season, daily variation in flower opening and pollen release can significantly alleviate pollination competition (Stone *et al.* 1998; Štenc *et al.* 2023). This temporal separation of flowering peaks, or daily partitioning of flower opening, can also enhance the pollination service for subsequent flowering species by gradually providing resources to pollinators, thus ensuring the maintenance of the local pollinator community (Botes *et al.* 2008; Sargent and Ackerly 2008; Mesquita-Neto *et al.* 2015).

The Brazilian Cerrado is a diverse tropical savanna characterized by the strong seasonality imposed by the sequence between the dry and the rainy seasons (Morellato *et al.* 2013). Phenology studies in the Cerrado have shown that different species flower throughout the year, with peaks at the end of the dry season and the beginning of the wet season (Batalha and Martins 2004; Morellato *et al.* 2013). It has been suggested that these differences may relate to seed dispersal timing, phylogenetic relationships, and pollinator availability in addition to specific climatic conditions (Sarmiento and Monasterio 1983; Oliveira and Gibbs 2000). Hence, the reproductive phenology of many Cerrado species follows the seasonal climate, with most species being outbreeders and strongly relying on animal pollinators to yield fruits and seeds (Oliveira and Gibbs 2000). Because the period for flowering usually coincides with the rainy season, many species in the Cerrado display overlapping flowering phenologies. For instance, Mesquita-Neto *et al.* (2015) found substantial flowering overlap and pollinator sharing in eight cooccurring *Psychotria* species with similar flower morphology. While flowering with congeneric species might increase floral abundance and attract more pollinators, it might also affect intra- and interspecific patterns of pollen delivery and deposition (Mesquita-Neto *et al.* 2018; Bergamo *et al.* 2020).

**Figure 1.**
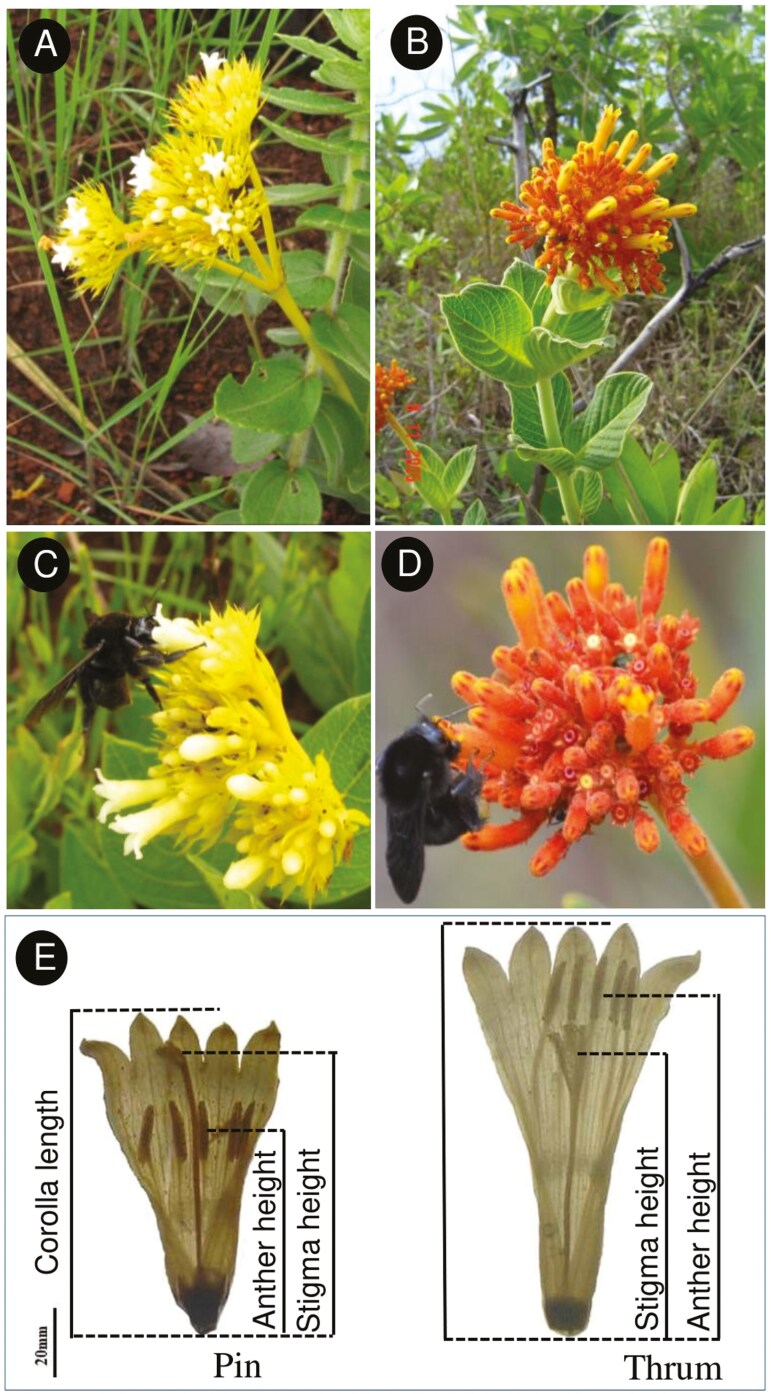
Palicourea coriacea (A) and *Palicourea officinalis* (B) flowers, *Bombus pauloensis* visiting flowers of both species (C, D) and traits measured on dissected photographed Pin and Thrum flowers of both species (E).

In the present study, we investigated the pollination ecology of *Palicourea coriacea*, a Rubiaceae species native to the Brazilian Cerrado, in relation to its flowering phenology and coflowering with congeneric *P. officinalis.* Flowers of both species are tubular, differing in colour, with *P. coriacea* flowers being pale yellow with yellow pedicels, while *P. officinalis* being dark yellow to orange with red pedicels (Fig. 1A–D). Both species exhibit distyly, a sexual polymorphism characterized by the presence of two floral morphs, Pin and Thrum (P and T hereafter, Fig. 1E), with reciprocal herkogamy and the typical heteromorphic incompatibility system, so seed production depends on pollen transfer between different morphs (Consolaro *et al.* 2009). The two species are visited primarily by bees (*P. coriacea*) and hummingbirds (*P. officinalis*) (Consolaro *et al.* 2009; Furtado *et al.* 2021). Field observations suggest that flowering in *P. coriacea* starts soon after the start of the rainy season and earlier than *P. officinalis* (Consolaro 2008). Here, we described differences in corolla size and reproductive organs, pollen morphology and production, and in nectar production and sugar concentration to identify if trait variation could relate to differences in the floral visitors and pollination syndrome. We also analysed differences in anther and stigma height to determine the potential for interspecific pollen transfer based on the similarity in organ height between species. Then, we investigated the pollination ecology of *P. coriacea* to describe patters of pollinator visitation and pollinator sharing with *P. officinalis*, pollen delivery and deposition on stigmas at two phenological times: early in the season, and then during the coflowering period with *P. officinalis*. We interpreted the results in the context of pollinator preference and sharing, and the consequences of flowering overlap for pollen dynamics.

## Material and methods

### Study site

The study was conducted in the IBGE Ecological Reserve, a protected area of 13 km^2^ hectares 35 km south of Brasilia (15º 55′–15º 58′ S and 47º 52′–47º 55′ W) within the Cerrado biome. The region’s climate is CW (Köppen classification), with a dry season from May to September and a rainy season from October to April. At an altitude between 1.000 and 1.150 m, the average rainfall is 1.550 mm, and the average temperature is 21°C (Consolaro *et al.* 2009). The study was conducted in area of ca. 0.25 km^2^ characterized by the vegetation type known as *Campo sujo,* consisting of herbaceous species with shrubs and small trees scattered sparsely throughout (Goodland 1971), including *P. officinalis* (Rubiaceae), *Psidium salutare* (Myrtaceae), *Erythroxylum suberosum* and *E. campestre* (Erythroxylaceae), *Vochysia thyrsoidea, Qualea parviflora* and *Q. multiflora (*Vochysiaceae*), Emmotum nitens* (Metteniusaceae*), Miconia albicans* (Melastomataceae*), Xylopa* sp. (Annonaceae*), Byrsonima sp. (*Malpighiaceae*), Chamaecrista* sp. (Fabaceae), *Tocoyena formosa,* and *Chomelia* sp. (Rubiaceae), as some of the common species that flowered during the study. At the IBGE Ecological Reserve, it is possible to find the close relatives of the study species *P. rigida* and *Psychotria carthagenensis* but these two species did not occur within the area used for the study.

### Flowering phenology

The flowering period of *P. coriacea* and *P. officinalis* was recorded in 40 and 20 individuals, respectively, on a transect of ca. 900 m, allowing at least 5 m between individuals of the same species. The goal was to detect the start of the overlap in the flowering of both species during the study period to conduct the pollination studies described below. Observations were made weekly between October and December 2015, recording the number of open flowers on each marked plant. The comparisons involved using the percentage of flowers open in the tagged individuals that showed a flowering phenophase (Bencke and Morellato 2002). Pianka index (Pianka 1973) was used to calculate the flowering overlap of the two species. The index ranges from 0 (no overlap) to 1 (total overlap), and is given by the equation:


$$\begin{array}{l} O_{12}=\;\frac{\overset{n}{\underset{i=1}{\sum }}P_{2i}\;P_{1i}}{\sqrt{\overset{n}{\underset{i}{\sum }}P_{2i}^{2}}\overset{n}{\underset{j}{\sum }}P_{1i}^{2}} \end{array}$$

where *O*_12_ = the overlap of flowering phenophase between species 1 and 2; while *P*_1*i*_ = proportion of flowers *i* according to the total of species 1; *P*_2*i*_ = proportion of flowers *i* according to the total of species 2. The value generated by the overlap is arbitrarily considered high (>0.6), intermediate (0.4–0.6) or low (<0.4) (Grossman 1986).

### Species comparison of the flower morphology, pollen, and nectar traits

Thirty-six to 184 flowers per species were collected (1–4 flowers per individual plant, including 81 (184 flowers) P and 17 (36 flowers) T plants of *P. coriacea* and 24 (64 flowers) P and 25 (67 flowers) T plants in *P. officinalis*) and fixed in 70% ethanol for measurements. In the laboratory, flowers were dissected and photographed, and the traits were measured using ImageJ 1.45s software (https://imagej.net/). The floral traits measured included corolla length, anther height, and stigma height in mm (Fig. 1E). The anther height and stigma height were measured from the base of the flower up to the middle point of the anther and stigmatic lobe.

To determine differences in pollen size between species, ten floral buds in pre-anthesis of each morph and species (one floral bud per individual plant) were collected and fixed in 70% alcohol. In the lab, one anther per floral bud was squashed on a microscope slide using glycerol jelly stained with fuchsin, and the extracted pollen grains were photographed under an optical microscope using 40× magnification (photographs were taken with a digital camera attached to a Bio.Labmb 210p-40p microscope). Pollen diameter from 10 pollen grains per sample was measured from the digital photos using ImageJ 1.45s software. To assess differences between species in pollen production, 14 to 19 floral buds in pre-anthesis of each morph per species (one floral bud per individual plant) were collected in the field and fixed in 70% alcohol. For each floral bud, one anther was removed and squashed on a microscope slide with a drop of acetic carmine to visualize and count all pollen grains under an optical microscope. Subsequently, the number of pollen grains per sample was multiplied by four (number of anthers per flower) to estimate the total number of pollen grains per flower.

The total nectar volume and sugar concentration were assessed separately for the morphs of the two species using 14 P and 11 T plants in *P. coriacea*, and 12 P and 6 T plants in *P. officinalis* (23–36 flowers for each morph per species; 1–9 flowers per individual plant). Inflorescences bearing floral buds 1 or 2 days before anthesis were bagged with tulle bags to prevent visitation. Measurements were completed the day flowers opened from 17:00 to obtain the total nectar volume (*Palicourea* flowers produce nectar only once and last one day, Consolaro, pers. obs.). The volume was measured using 10 μl microcapillary tubes (Hirschmann Laborgeräte GmbH & Co. KG), and the sugar concentration was measured using a pocket refractometer (Digit, model 107BP). Differences in floral traits, pollen size, nectar volume, and sugar concentrations were compared using a linear mixed effect model including species, morph, and the interaction term (LMMs). The analyses of floral traits (corolla length, anther height, and stigma height) and nectar traits included individual plants as a random factor since 1 to 9 flowers were used to obtain the data. For pollen diameter, the term flower was included as a random factor since repeated measures were obtained from anthers of the same flower. The LMMs were done using the ‘lmer’ function in the ‘lme4’ package (Bates *et al.* 2023). The ‘lsmeans’ function with a Tukey adjustment implemented using ‘cld’ function from the package ‘multcomp’ (Piepho 2004) was used as a post hoc test to detect significant differences between morphs within and between species. Pollen production data was analysed with a linear model (LM) using the ‘lm’ function. A generalized linear model (GLM) with a Poisson distribution implemented using the ‘glm’ function in the ‘lme4’ package was also conducted for pollen production. Subsequently, a comparison was conducted between the models (LM and GLM) for pollen production, using ‘anova’ function and test ‘Chisq’, providing the best fit for the LM (results not shown). Thus, the analyses proceeded with the best model. For all models, the ‘Anova’ function was used to obtain the significance of each factor and the ‘lsmeans’ and ‘cld’ functions to perform the post hoc test, verifying differences between morphs within and between species. All analyses described here and below were performed using R (R Core Team 2024).

### Floral visitors, pollen removal, and deposition under natural conditions

**Figure 2.**
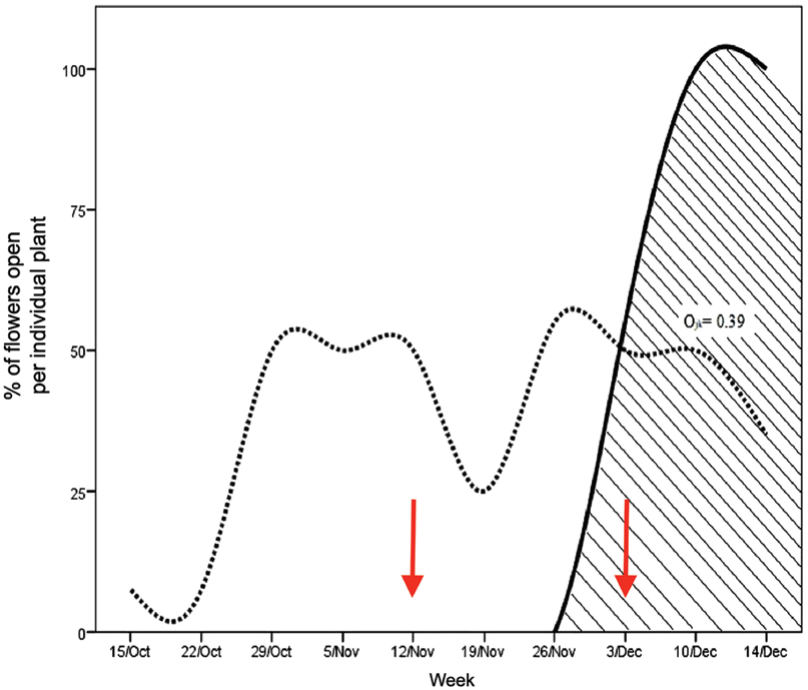
Flowering phenology of *Palicourea coriacea* (dashed line) and *Palicourea officinalis* (solid line) during the study period, totalling 10 weeks, indicating the the percentage of flowers open in the tagged individuals that were flowering during the study. The two arrows indicate the periods in which pollinator observations and flower collection for pollen delivery and deposition on the stigma were conducted.

The collection of these data was done in the proximities of the plants used to obtain flowering phenology data, using different plants to those marked for phenology data collection. Pollinator observations and subsequent collection of flowers for pollen removal and deposition on stigmas were conducted at two different periods. The first period was 4 weeks after the start of flowering of *P. coriacea*, before the initiation of *P. officinalis* flowering (early period hereafter). The second was ca. 10 days after the initiation of flowering in *P. officinalis* (coflowering period hereafter, see Fig. 2). During the coflowering period, the pollinator observations and flower collection were conducted in patches with only *P. coriacea* plants (hereafter conspecific patches) and in patches where both species flowered together (hereafter congeneric patches), the later noting what *Palicourea* species received visits. All observations during the two phenological periods were conducted in patches of ca. 1.5 × 1.5 m with 5 to 10 stems and during two days per period (since the vegetation was dense, it was not possible to distinguish individual plants but stems with inflorescences). In the second period, coflowering patches were selected ensuring that the two species were equally represented per patch (2–3 or 4–5 stems per species). Observations were conducted by 3 to 4 observers between 7:00 and 15:00 in 15-min intervals, writing the floral visitor to the lowest possible taxonomic level and the number of visits. After completion of the 15 min. observation, each observer moved to a new patch at least 2 m apart from the previous one. Since the study area was large (this is, 0.25 km^2^), it was possible to avoid using the same patch by multiple users or at different times, and all observers were at least 10 m distant apart each. Hence, each patch was observed only once. All observers randomly selected patches and used both conspecific and congeneric patches. In total, observations were conducted in 159 patches during the early period, and in 196 patches during the coflowering period (100 conspecific and 96 in congeneric), accumulating 39.75 and 49 h of observation respectively. Insects were collected for identification, while hummingbirds were identified during the observations. The insects collected were deposited at the Integrated Zoology and Botany Laboratory of the Federal University of Goiás Catalão Region (Brazil). *Bombus pauloensis* (formerly known as *B. atratus*) was the most common floral visitor (see results below), and the length of the glossa was measured in the sampled individuals.

During the pollinator censuses, observers collected open flowers (*Palicourea* flowers only last one day) exposed to pollinators. The first collection was done at 7:00 (sampling flowers outside the targeted patch for observation), and then every 2 h after completion of the pollinator observation (this is after the 9:00, 11:00, 13:00, and 15:00 interval) sampling visited flowers to measure pollen delivery (quantified as pollen left in the anthers) and pollen deposition on the stigmas. In total, 2 to 14 flowers were sampled per patch, totalling 167 flowers in 25 patches during the early period (15 patches between 7:00 and 11:00, 10 patches between 13:00 and 15:00), and 243 flowers in 30 patches during the coflowering period (15 patches for conspecific and congeneric patches, 9 between 7:00 and 11:00, and 6 between 13:00 and 15:00). Since each patch was observed only once, and the collection of flowers was done after observations, we avoided the potential effect of the removal of flowers in a patch on the pollinator visitation and therefore on pollen movement. With this sampling strategy, it is possible to infer if flowers collected later in the day present more pollen on their stigmas or less pollen in their anthers, as expeced after repeated visits by pollinators to the same patch and flower, and evidence of pollinator preference if differences arise when comparing conspecific and congeneric patches. Flowers were stored in individual vials and frozen at −20°C until processed. In the laboratory, flowers were dissected under the microscope. Styles were cut at the base, close to the ovary. All the anthers and stigmas were mounted independently on glycerine with safranine. Then, all pollen grains left in the anthers and the pollen on the stigma were counted under the 40× objective in the microscope. The preparation of samples and pollen count was done by the same person to avoid any bias in the data associated with multiple participants on the data collection.

Analyses on pollinator visitation focussed on *B. pauloensis* since this pollinator conducted more than 90% of the visits (see results below, Table 1). All the analyses on pollinator visitation were done using Generalised Linear Mixed Models (GLMMs), including a number of visits observed in a patch (corresponding to an observation interval) as response variable modelled according to a negative binomial distribution using the function glmmTMB and the family nbinom2 from the package ‘glmmTMB’ (Brookes *et al.* 2017). Then, we used the ‘Anova’ function as described above. The first analysis compared *B. pauloensis* visits between the early and the coflowering period (flowering period as predictor) using only the data collected for *P. coriacea*. In the second analysis, using the data obtained during the coflowering period, the same approach was used with patch type (PT) as a predictor to compare visitation to *P. coriacea* in conspecific and congeneric patches. Finally, the data obtained in conspecific patches was used to compare *Bombus* visitation between *P. coriacea* and *P. officinalis;* hence, the term species was set as a predictors in the analysis. All these models included the ‘observer’ as a random factor to account for the random variation associated with the patches selected by observers (we did not include patch as random factor since the unit of observation was number of visits at the patch level).

**Table 1.** Total number of pollinator visits during the observations conducted in the early period, when *Palicourea coriacea* flowered before *Palicourea officinalis*, and during the coflowering period in conspecific patches, and congeneric patches with *P. officinalis*. The observations of the coflowering period indicate the number of visits of *Bombus pauloensis* and *Colibri serrirostris* to *P. coriacea* (first value) and to *P. officinalis* (second value). The rest of observations correspond to P. coriacea.

Period of observation	Patch type	Floral visitor	Total number of visits	Percentage (%)
Early period (before flowering of *P. officinalis*)	NA	*Bombus pauloensis*	638	91.93
		*Centris* sp	35	5.04
		Moph sp1	4	0.58
		Moph sp2	13	1.88
		Moph sp3	4	0.57
Coflowering period	Conspecific patch	*Bombus pauloensis*	1604	99.13
		*Centris* sp	5	0.31
		Lepdoptera	8	0.49
		*Toxomerus* sp	1	0.06
Coflowering period	Congeneric patch	*Bombus pauloensis*	860 (473, 387)	93.66
		*Centris* sp	3	0.32
		Halictidae sp1	3	0.32
		*Colibri serrirostris*	59 (24, 35)	6.33
		*Phoebis* sp	1	0.11
		Vespidae sp1	5	0.54

The data on pollen delivery (measured as the pollen grains left in anthers, see Material and Methods above) and pollen deposition on the stigmas was analysed separately for P and T flowers since the patterns of pollen pick up and deposition depicted by bees and hummingbirds in *Palicourea* species can vary according to the floral morph visited (Raupp *et al.* 2020; Furtado *et al.* 2021, 2023). In addition, it was not possible to distinguish the pollen species identity due to the overlap in pollen grain size between species and morphs (see results below). The analyses entailed used of GLMMs to compare pollen delivery or deposition on the stigmas modelled according to a negative binomial distribution as described above. These analyses compared pollen patterns between the early and the coflowering period (with no distinction between conspecific and congeneric patches for the latter). Then, using only the data collected during the coflowering period, the comparisons of pollen delivery and deposition were done between PT (i.e. conspecific vs. congeneric). All the analyses included the factor sampling time and the interaction term (using the predictor ‘flowering period’ or ‘PT’ depending on the model), grouping the intervals of sampling time into samples collected in the morning, between 7:00 and 11:00, and those collected in the afternoon, between 13:00 and 15:00 (see justification above). In all analyses, the unit of observation was the data collected at the flower level. Hence, since several flowers were sampled per patch, patch was included as a random factor in all the models.

## Results

### Flowering phenology

*Palicourea coriacea* and *P. officinalis* started flowering after the second week of October and the end of November, respectively, with a flowering interval of 5 weeks where *P. coriacea* flowered alone (Fig. 2). For *P. coriacea*, individuals displayed 25%–50% flowers opened, while in *P. officinalis* flower opening was fast and individuals presented more than 50% of flowers open. During the study period, the flowering of the two species overlapped by 0.4, demonstrating certain synchrony in the flowering period, according to the Pianka index. The magnitude of the overlap was probably underestimated since the phenology data collection concluded before the end of the flowering season for the two species (Fig. 1).

### Species comparison of the flower morphology, pollen, and nectar traits

There were differences in corolla length and the height of the anthers and stigmas between species and morphs (Table 2). The interaction term species × morph was statistically significant only for stigma height (Table 2). *Palicourea coriacea* flowers present, on average, shorter corollas (mean ± s.d. (*n*):10.35 ± 2.57 (220)) than *P. officinalis* (mean ± s.d.:13.08 ± 1.53(131)), with no differences at the morph level, so that, on average P and T flowers within species have similar corolla length (Table 3). For both species, differences appeared when comparing anther height and stigma height between morphs. Specifically, P flowers showed taller stigmas and shorter anthers, while T flowers present anthers above the stigma, hence confirming the typical floral architecture of distylous species. Average anther height was, in general, taller in *P. officinalis* than *P. coriacea* for P and T morphs. The opposite was found for stigma height so *P. coriacea* displayed greater stigma height values for both morphs (see Table 3 for average organ length per species). Interestingly, a visual comparison of reciprocal organs shows that the average height of tall organs (T anther and P stigma) is more similar between species than within species. The same pattern occurs for small organs, so the average T stigma height is closer to the average P anther height between species than within species (Table 3). For example, the average anther height of T *P. coriacea* flowers (12.44 ± 1.52) is closer to the average stigma height of P *P. officinalis* (12.71 ± 1.25) than *P. coriacea* flowers (13.46 ± 1.59). This means that the two species possess similar organ height architecture which could facilitate pollen transfer between species.

**Table 2.** Results of the analysis to evaluate the differences between species, floral morphs, and the interaction effect on floral traits, and the characteristics of pollen grains and nectar in *Palicourea coriacea* and Palicourea officinalis^*^.

Flower traits	Source of variation					
	Species		Morph		Species *Morph	
	*F*	*P*-value	*F*	*P*-value	*F*	*P*-value
Floral morphology						
Corolla length	33.51	<.001	7.94	<.01	0.03	.87
Anther height	20.18	<.001	76.48	<.001	1.87	.17
Stigma height	31.34	<.001	162.38	<.001	11.92	<.001
Pollen						
Size	0.22	.64	18.83	<.001	0.02	.90
Production	20.16	<.001	0.34	.56	0.01	.91
Nectar						
Volume	0.004	.95	0.01	.94	0.51	.48
Concentration	66.74	<.001	0.36	.55	0.01	.93

^*^See Table 3 for the sample size and estimated mean ± SD for each trait.

**Table 3.** Measurements (mean ± SD) of the floral traits of Thrum and Pin flowers in *Palicourea coriacea* and *Palicourea officinalis*. Different letters represent significant differences (≤ 0.05) between morphs and species. The number of flowers or pollen grains (for size) used for each measurement is presented in parentheses.

Flower traits	*Palicourea coriacea*		*Palicourea officinalis*	
	Thrum	Pin	Thrum	Pin
Floral morphology				
Corolla length (mm)	11.27 ± 2.28ab (36)	10.17 ± 2.60a (184)	13.72 ± 1.55c (67)	12.42 ± 1.21bc (64)
Anther height (mm)	12.44 ± 1.52b (36)	9.98 ± 1.78a (184)	14.20 ± 1.60c (67)	11.00 ± 0.92b (64)
Stigma height (mm)	11.07 ± 1.45b (36)	13.46 ± 1.59c (184)	8.48 ± 1.10a (67)	12.71 ± 1.25c (64)
Pollen				
Size (μm)	69.14 ± 4.68b (100)	63.69 ± 6.38a (100)	68.69 ± 5.65b (100)	62.92 ± 5.11a (100)
Production (pollen count)	2040.53 ± 545.74b (19)	2134.69 ± 450.42b (16)	1435.00 ± 407.36a (14)	1498.57 ± 729.67a (14)
Nectar				
Volume (µl)	3.20 ± 0.99a (23)	3.12 ± 1.43a (36)	3.53 ± 1.32a (30)	3.36 ± 1.62a (32)
Concentration (% sugar)	23.19 ± 2.29b (23)	24.18 ± 2.27b (36)	14.63 ± 5.56a (30)	14.77 ± 5.51a (32)

Thrum pollen grain was larger than P pollen, but the two species showed similar pollen grain size (Table 2 and 3). This prevents the distinction of species identity on pollen grain counts on stigmas. The analyses on pollen production revealed general differences between species, with *P. coriacea* (2083.57 ± 499.45) producing more pollen than *P. officinalis* (1466.79 ± 580.77), while pollen production was similar between morphs (Table 3), a consistent pattern for the two species (Table 2).

Nectar volume was similar between species and morphs (Tables 2 and 3). For the concentration of sugar in the nectar, there were significant differences between the species (Table 2), with *P. coriacea* (23.79 ± 2.31%) producing nectar with a higher concentration than *P. officinalis* (14.70 ± 5.49%). No significant differences were found between morphs or the interaction term with species (Table 2). Hence, both morphs of *P. coriacea* have nectar with a higher sugar concentration than that of *P. officinalis* (Table 3).

### Floral visitors, pollen removal, and deposition under natural conditions

The main floral visitor of *P. coriacea* at the study site, both in the early and the coflowering period with *P. officinalis*, was *B. pauloensis* (Table 1). The visits consisted of legitimate visits to harvest nectar (we did not observe a nectar-robbing behaviour). During the early period, 91.93% of the visits were conducted by *B. pauloensis*, while observations of residential hummingbirds (*Eupetomena macroura* and *Colibri serrirostris*) were anecdotal and outside the formal censuses. During the coflowering period and in congeneric patches with the two *Palicourea* species, 93.66% of the visits were conducted by *B. pauloensis*, which visited the two species. *Colibri serrirostris* was only observed in the formal censuses of congeneric patches, representing 6.33% of all visits and visiting both *Palicourea* species. In contrast, 99.1% of the visits to *P. coriacea* in conspecific patches were conducted by *B. pauloensis*. The mean ± s.e (*n*) probiscid length of *B. pauloensis* in the study population is 6.2 ± 0.3 (20) mm.

**Figure 3.**
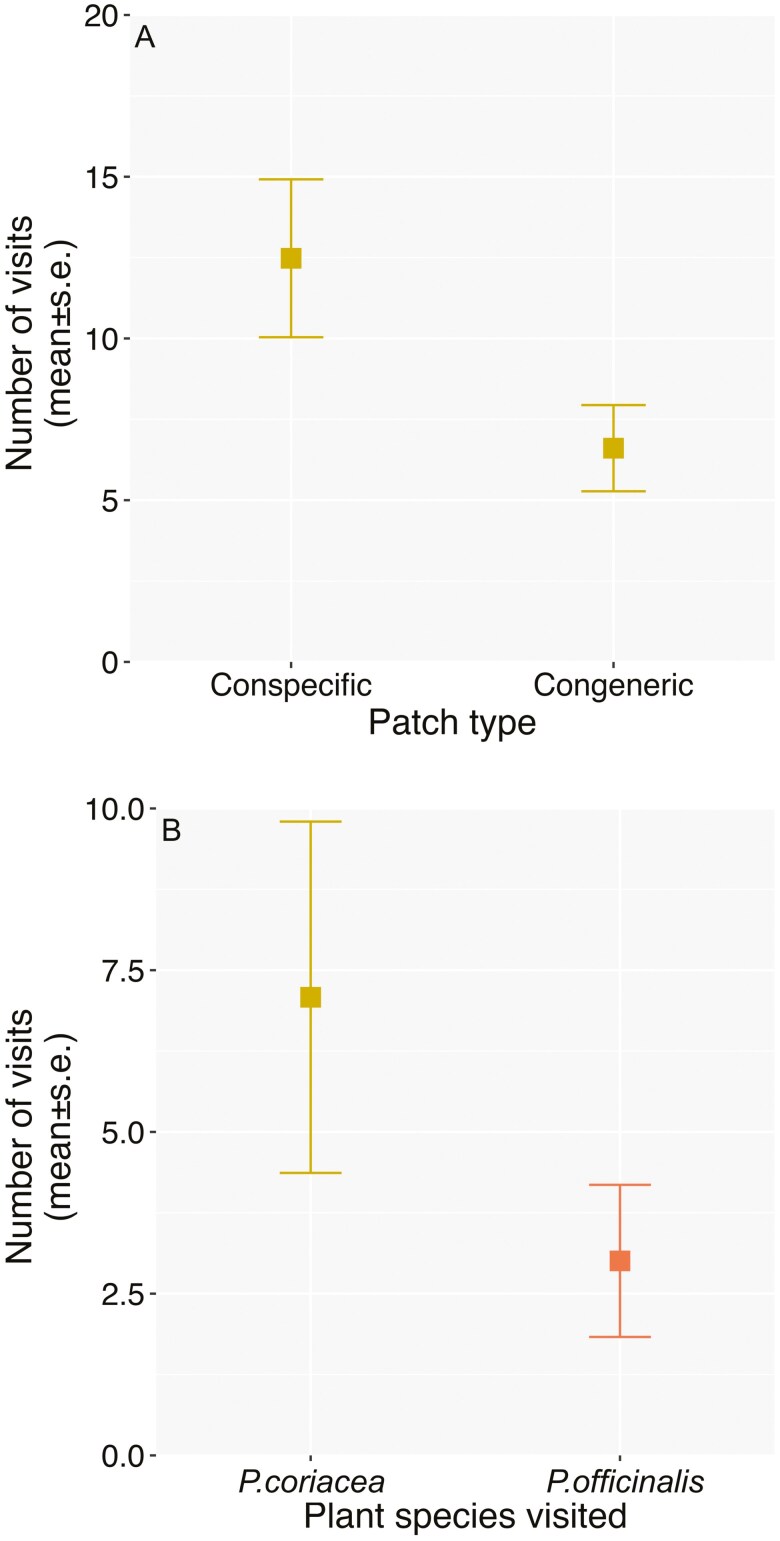
Estimates and s.e derived from the GLMMs on the number of visits by *Bombus pauloensis* to (A) *Palicourea coriacea* during the coflowering period in conspecific patches and congeneric patches with *Palicourea officinalis*, and (B) in congeneric patches with the two species to *P. coriacea* and *P. officinalis*.

The comparison of *Bombus* visitation between the two flowering periods revealed higher visitation on *P. coriacea* during the coflowering period (χ²= 14.675, d.f.=1, *P* = .003; mean ± s.e.(*n*) of visits during early period: 2.98 ± 0.97 (158), coflowering period: 7.77 ± 2.72 (194)). The visitation of *B. pauloensis* to *P. coriacea* during the coflowering period was higher in the patches with only *P. coriacea* (12.48 ± 2.44 (102)) than the patches with the two species (6.61 ± 1.33 (91); χ²=10.84, d.f.=1, *P* = .001, Fig. 3A). Finally, the comparison to detect if *B. pauloensis* visitation differed between species revealed higher visitation in *P. coriacea* (7.08 ± 2.71(91)) than *P. officinalis* (3.01 ± 1.18 (91); χ²=7.5917, d.f.=1, *P* = .006, Fig. 3B). Supplementary Tables S1–S3 include the GLMMs estimate values of the intercept, fixed factor and random effect of the pollinator visitation models presented.

**Figure 4.**
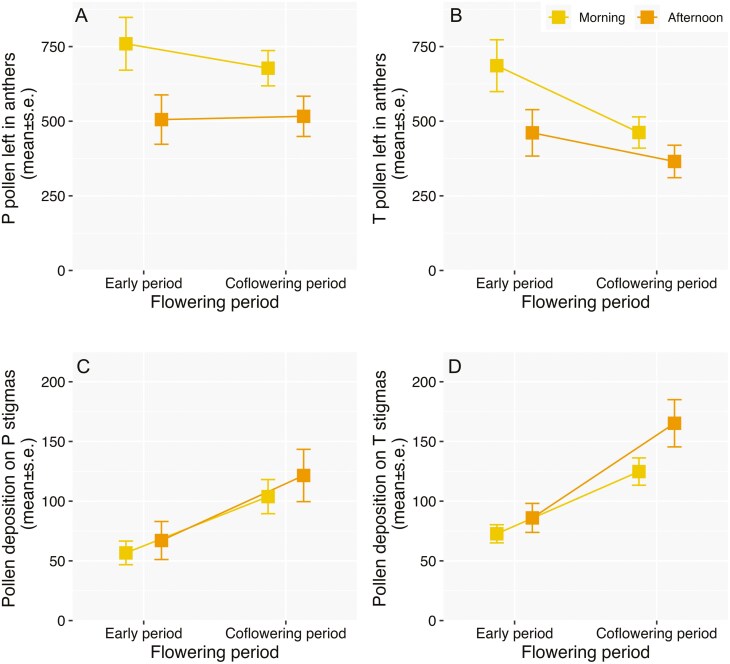
Estimates and s.e derived from the GLMMs of pollen delivery (measured as pollen left in anthers) and pollen deposition on the stigmas in *Palicourea coriacea* flowers sampled along the flowering season, before (early period) and once *P. officinalis* bloomed (coflowering period), representing Pin (A) and Thrum (B) pollen counted in anthers, and pollen arrival on Pin (C) and Thrum (D) stigmas, in flowers sampled in the morning (between 7 and 11 a.m.) and in the afternoon in (between 13 and 15 p.m.). The summary results, including the estimates for the intercept, predictors and random effect are reported in Supplementary Tables S4 and the values used for the plot in Supplementary Table S5.

Pollen delivery (measured as pollen left in anthers) and deposition on the stigmas displayed different patterns in the two periods of the study. Pollen delivery of P flowers was similar in the two periods (Fig. 4A, Table 4, and Supplementary Table S5), while pollen delivery of T flowers was higher during the coflowering period (less pollen left in the anthers) than the early flowering period (Fig. 4B, Table 4, and Supplementary Table S5). In general, there was a trend of less pollen left in the anthers of the flowers sampled in the afternoon than in the morning (Fig. 4A and B, Table 4, and Supplementary Table S5). Specifically, the amount of pollen left in the anthers of P flowers ranged between 35.6% and 23.7% for the morning and afternoon sampling intervals in the early period, and 31.7% and 24.2% for the coflowering period. For T flowers, the estimates represent 33.6% and 22.6% for the early period, and 22.7% and 17.9% for the coflowering period (% obtained using the average pollen production for P and T in Table 3 as reference for pollen per flower, and the predicted estimate of pollen counts in anthers according to the GLMMs in Supplementary Table S5). For both P and T flowers, the amount of pollen that reached the stigmas during the coflowering period was ca. twice as large as in the early period, and no substantial differences regarding the time in which flowers were sampled (Table 4 and Supplementary Table S5, Fig. 4C and D; see Table S5 for the predicted estimates of pollen counts on stigmas). Taken together, these results indicate that the general higher visitation during the coflowering period most likely resulted in higher rates of pollen delivery and deposition. However, the time of flower sampling (morning vs. afternoon) only made a difference for pollen delivery, suggesting that repeated visits through the day to the same flower benefit pollen export.

**Table 4.** Results of the GLMM to compare the patterns of pollen delivery (measured as pollen left in anthers) and deposition on the stigmas of Pin and Thrum flowers of *Palicourea coriacea* during the two flowering periods (P) used (early period, when the species flowered solitarily, and coflowering period with *P. officinalis*), including the variation associated with the sampling time (S) within the day (samples collected in the morning between 7:00 and 11:00, and afternoon between 13:00 and 15:00). Supplementary Table S4 includes the GLMMs estimate values of the intercept, fixed factor, and random effect of the models presented.

Pollen delivery factors	Pin anthers		Thrum anthers	
	χ²	*P*-value	χ²	*P*-value
Flowering period (P)	0.6082	.44	5.4081	.02
Sampling time (S)	4.0902	.04	3.5530	.06
P × S	0.2902	.59	0.3304	.57

Pollen deposition factors	Pin stigmas		Thrum stigmas	
	χ²	*P*-value	χ²	*P*-value
Flowering period (P)	7.4677	.006	15.0412	.0001
Sampling time (S)	0.3298	.57	0.9309	.34
P × S	0.0009	.98	0.2399	.62

**Figure 5.**
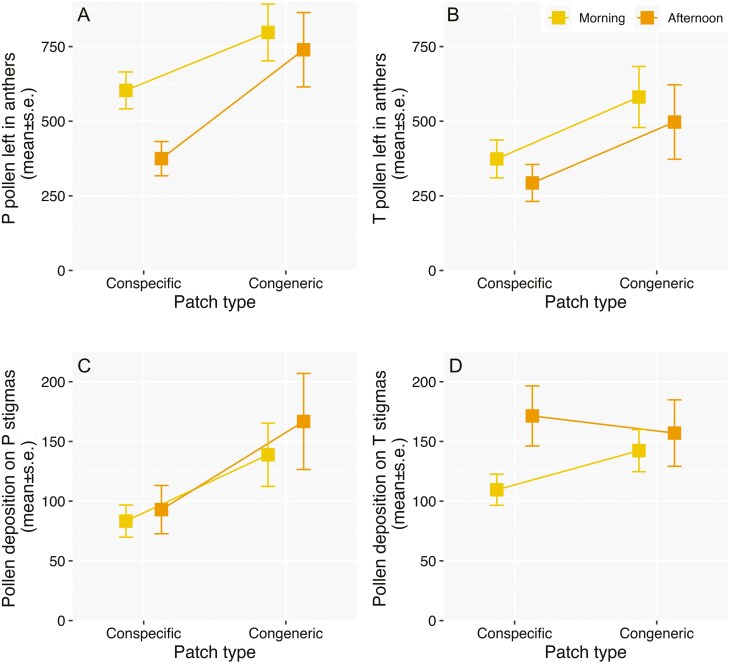
Estimates and s.e derived from the GLMMs of pollen delivery (measured as pollen left in anthers) and pollen deposition on the stigmas in *Palicourea coriacea* flowers sampled during the coflowering period, in conspecific patches and in congeneric patches with *P. officinalis*, representing Pin (A) and Thrum (B) pollen counted in anthers, and pollen arrival on Pin (C) and Thrum (D) stigmas, in flowers sampled in the morning in (between 7 and 11 a.m.) and in the afternoon in (between 13 and 15 p.m.). The summary results, including the estimates for the intercept, predictors and random effect are reported in Supplementary Tables S6 and the values used for the plot in Supplementary Table S7.

The comparisons during the cofloweing period revealed a trend of less pollen in the anthers of P and T flowers of *P. coriacea* in conspecific patches compared to congeneric patches with *P. officinalis,* with similar patterns in the morning and afternoon sampling period (Table 5 and Supplementary Table S6, Fig. 5A and B). For conspecific patches of *P. coriacea*, this translates in 28.3% and 17.5% pollen left in P anthers in the morning and afternoon sampling time, compared to the 31.7% and 24.2% in congeneric patches with *P. officinalis*. For T flowers, the % of pollen left in anthers in conspecific patches represents 18.3% and 14.4% in the flowers sampled in the morning and afternoon, while in congeneric patches these values are 28.5% and 24.4% (see Supplementary Table S7 for predicted estimates of pollen counts in anthers derived from the GLMMs in Supplementary Table S6). These findings seem to indicate that the higher visitation of *B. pauloensis* to *P. coriacea* flowers in conspecific patches results in more pollen exported. For pollen counts on the stigmas, it was found that P stigmas in congeneric patches with *P. officinalis* received more pollen than in conspecific patches, while pollen deposition patterns for T flowers were similar in the two patch conditions (Table 5 and Supplementary Table S6, Fig. 5C and D; see Supplementary Table S7 for the predicted estimates of pollen counts on stigmas).

**Table 5.** Results of the GLMM to investigate the patterns of pollen delivery (measured as pollen left in anthers) of Pin and Thrum flowers of *Palicourea coriacea* during the coflowering period, according to the PT (conspecific patches of *P. coriacea* and congeneric patches with *P. officinalis)*. The analyses incorporated the variation in patterns of pollen delivery according to the sampling time (S) within the day (samples collected in the morning between 7:00 and 11:00, and afternoon between 13:00 and 15:00). Supplementary Table S5 includes the GLMMs estimate values of the intercept, fixed factor, and random effect of the models presented.

Pollen delivery factors	Pin anthers		Thrum anthers	
	χ²	*P*-value	χ²	*P*-value
Patch type (PT)	3.1563	.08	3.2570	.07
Sampling time (S)	0.1339	.71	0.2584	.61
PT × S	2.100	.15	0.0451	.83

Pollen deposition factors	Pin stigmas		Thrum stigmas	
	χ²	*P*-value	χ²	*P*-value
Patch type (PT)	4.1924	.04	2.3351	.13
Observation period (O)	0.3564	.55	0.2059	.65
PT × O	0.0326	.86	1.4820	.22

## Discussion

Our observations of flowering phenology revealed that *P. coriacea* began flowering in the second half of October, maintaining its blooming approximately a month before *P. officinalis* began to flower. The rate of flowering in *P. coriacea* was slower, as indicated by the average percentage of open flowers per inflorescence. Due to logistic limitations in tracking the full flowering season, it is likely that we underestimated the extent of flowering overlap and synchrony between the two species. Nonetheless, our findings align with regional data for these species. Using herbarium records, Consolaro (2008) reported that *P. coriacea* flowers from July to March (although in the study population flowering ends in January, Consolaro, pers. obs.), while *P. officinalis* flowers between October and January. These phenological patterns are consistent with those observed in other sympatric species in the Brazilian Cerrado and other seasonal ecosystems, where the onset of the main flowering season corresponds with the arrival of the rainy season, and species’ flowering begins sequentially (Madeira and Fernandes 1999; Botes *et al.* 2008; Ramos *et al.* 2014; Mesquita-Neto *et al.* 2015). However, our data derives from a single sympatric locality and one season, so the adaptive significance of sequential flowering to reduce pollinator sharing remains an open question. Flowering is a phylogenetically conserved trait (Kochmer and Handel 1986; Strauss *et al.* 2021; Park *et al.* 2022), and differences in flowering might not be necessarily evolved in response to avoidance of pollinator competition. Nevertheless, the goal of our study was to identify two distinct flowering periods, early flowering and flowering with conspecifics, and this offered valuable insights into the pollination and reproductive ecology of *P. coriacea* in relation to a closely related species that share the same pollinators.

### Floral trait species variation and floral visitors

The two species under study are shrubs presenting flowers grouped in dense inflorescence (Fig. 1A and B), with typical tubular flowers characteristic of the genus *Palicourea*. Species differ in that *P. coriacea* presents pale yellow flowers with shorter corolla tubes (mean ± s.e.:10.35 ± 0.17 mm), producing more pollen and nectar with higher sugar concentration, than the dark yellow to orange flowers of *P. officinalis* with longer corollas (13.09 ± 0.13 mm), lower pollen production and more diluted nectar (Table 3). These trait differences agree with a floral syndrome for bee pollination in *P. coriacea* (shorter corollas, higher sugar concentration), and hummingbirds in *P. officinalis* (Baker and Baker 1983; Frankie *et al.* 1983; Roubik *et al.* 1995; McDade and Weeks 2004). Previous pollination studies conducted at the same locality have shown that the primary visitors for *P. coriacea* and *P. officinalis* are bees and hummingbirds respectively, but receive visits, albeit less frequently, from other pollinator groups (Consolaro *et al.* 2009; Furtado *et al.* 2021). In our study, the main floral visitor for both species was *B. pauloensis*, but hummingbird visits were observed during the coflowering period in patches where the two *Palicourea* species cooccurred. Due to the lack of observations in conspecific patches of *P. officinalis* or extended observations throughout the rest of the season, we were unable to fully understand if the two *Palicourea* species partition functional pollinator groups, or detect changes in the relative contribution of the two pollinator groups as flowering progresses (Armbruster and Herzig 1984; Wolfe and Sowell 2006; Raine *et al.* 2007; Botes *et al.* 2008; Boas *et al.* 2013; Souza *et al.* 2017). Previous research has demonstrated the efficiency of hummingbirds and bees in the pollination of *Palicourea* species and their close relatives (Stone 1995, 1996; Lau and Bosque 2003; Ornelas *et al.* 2004; Valois-Cuesta *et al.* 2011; Furtado *et al.* 2023), which deserve future work in the species here studied.

Another important axis of trait differentiation among coflowering species involves differences in the spatial distribution of reproductive organs, a concept referred to as the sexual architecture hypothesis by Murcia and Feinsinger (1996). These authors proposed that coflowering species sharing pollinators and having similar sexual organ architecture are likely to experience increased interspecific pollen transfer. In the absence of different pollinators, even small changes in the architecture of reproductive organs could significantly reduce interspecific pollen transfer (Armbruster *et al.* 1994; Muchhala and Potts 2007; Norton *et al.* 2015; Eisen and Geber 2018; Newman and Anderson 2020). The distylous morphology of the studied species requires pollinators to promote legitimate pollination (i.e. pollen transfer between different morphs) for successful seed production (Consolaro *et al.* 2009; Furtado *et al.* 2021, 2023). In distylous species, legitimate pollination can be predicted by the accuracy of reciprocity, which is informed by the spatial match (particularly in reproductive organ height) between reciprocal tall (P stigmas and T anthers) and small organs (T stigmas and P anthers), and how pollinators interact with flowers to transfer pollen between morphs (Armbruster *et al.* 2006, *et al.*2009a; Furtado *et al.* 2023; Pérez-Barrales and Armbruster 2023). The species differed in the average height of anthers and stigmas in P and T flowers, but the spatial match between reciprocal tall and small organs was greater between species than within species. The overlap in the sexual architecture of opposite morphs between species, as described in other coflowering species including *Palicourea* (Cardoso *et al.* 2022), *Primula* (Kálmán *et al.* 2004; Keller *et al.* 2012), and *Linum* (Pérez-Barrales and Armbruster 2023), could lead to interspecific pollen transfer, which may incur reproductive costs (Morales and Traveset 2008; Arceo-Gómez and Ashman 2011; Moreira-Hernández *et al.* 2023; Pérez-Barrales and Armbruster 2023). The lack of discernible pollen differences between *P. coriacea* and *P. officinalis* made it difficult to accurately identify pollen specimens. The use of fluorescent dyes or quantum dots presents a promising method for quantifying pollen movement between species and obtaining reliable estimates of reproductive interference linked to pollinator sharing (Minnaar and Anderson 2019; Anderson and Minnaar 2020; Moir and Anderson 2023).

### Patterns of *Bombus pauloensis* visitation, pollen delivery, and deposition patterns

The bee community and bee abundance in the Brazilian Cerrado exhibit a seasonal rhythm, largely driven by the flowering phenology of the community and nectar availability, which fluctuate between the dry and wet seasons (Rabeling *et al.* 2019; Ballarin *et al.* 2022). Typically, bee activity decreases during periods of low rainfall and high temperatures (Truylio and Harter-Marques 2007). In the study site, Capellari (2011) described *B. pauloensis* as an important pollinator at the community level. In a later paper, the same author observed seasonal variations in the plant-pollinator network (which included *B. pauloensis*) that were dependent on the availability of floral hosts (Rabeling *et al.* 2019). Many social bees, including *B. pauloensis*, behave as aseasonal pollinators (Cameron and Jost 1998), functioning as keystone species by exploiting available floral resources throughout the year. Hence, it is possible that the increased visitation by *B. pauloensis* during the coflowering period might be driven by an increase of flowering species as the flowering season in the study area progressed. However, this might have created positive feedback in the pollination of *P. coriacea* for the following reason. While we did not monitor the flowering phenology of the entire community, our pollinator data revealed that (i) *B. pauloensis* was the primary visitor, accounting for over 90% of visits during both observation periods, and (ii) the visitation rate of *B. pauloensis* doubled between the two observation periods, which were approximately one month apart. When we focussed on the visitation data of the coflowering period, we detected two-fold higher visitation to *P. coriacea* in conspecific patches. In congeneric patches, *B. pauloensis* conducted twice as many visits to *P. coriacea* compared to *P. officinalis*. Taken together, these results indicate that, as the flowering season progressed, *B. pauloensis* increased its floral constancy on *P. coriacea*. Indeed, *B. pauloensis* is known for its relatively high constancy in visitation (Rossi *et al.* 2015). However, what remains unknown is the underlying mechanism that drives different visitation patterns during the coflowering period. Bumblebees are known to forage on plant species whose corolla tube length closely matches their proboscis length, allowing them to efficiently access nectar (Inouye 1978; Pyke 1982; Harder 1983, 1985; Soltz 1987; Pyke *et al.* 2012). In our study, the average proboscis length of *B. paoulensis* was 6.2 mm. So, when bumblebees reached a conspecific patch of *P. coriacea*, they probably fitted better the corolla and reached the nectar more easily, since the average corolla length encountered in a *P. coriacea* patch is closer to the proboscis length, finding more sugary nectar. In contrast, the uncertainty to find nectar in conspecific patches was probably higher, depending on whether bumblebees visited *P. officinalis*, with longer corollas and more diluted nectar, or *P. coriacea*. Bumblebees are known to respond rapidly to short-term changes in nectar availability, or change floral host depending on availability of rewards, preference, and previous experience (Jennersten *et al.* 1988; Flanagan *et al.* 2011; Natalis and Wesselingh 2012). The striking differences in visitation between patches with one or two species, suggests that this could be the case in our study, and deserves future work.

The patterns of bumblebee visitation across the two flowering periods corresponded, in general, with different pollen removal patterns, depending on the floral morph. Although pollen removal of P flowers was similar between the early and the coflowering period, T flowers experienced higher removal during the coflowering period. The analysis of the pollen data during the coflowering period showed that the flowers sampled in *P. coriacea* conspecific patches, which experienced higher visitation, had less pollen in the anthers, with stronger differences for T flowers (16% and 26% pollen left in anthers in conspecific and congeneric patches), that P flowers (23% and 28%). Taken together, these results suggest that the increase in visitation along the season, and the higher visitation in conspecific *P. coriacea* patches corresponded with higher pollen removal rates. This, in turn, might affect male fitness, at least in the likelihood of pollen export, and possibly seed siring. Our visitation data is per patch (not per flower), while the pollen data is per flower. Hence, we lack information on whether the sampled flowers received a single or multiple visits, so the link between the average visitation and average pollen removal is indirect. Despite this limitation, our results agree with experimental studied showing that flowers experiencing two or more visits by bumblebees have more pollen removed from anthers, which could lead to higher male fitness (Harder 1983; Galen 1992; Harder and Wilson 1994; Richards *et al.* 2009).

The pollen deposition values observed throughout the flowering season and across different patch types were consistent with those reported for other *Palicourea* species visited by hummingbirds (Ornelas *et al.* 2004; Valois-Cuesta *et al.* 2012; Raupp *et al.* 2020), bees (Wesselingh *et al.* 2000), or both (Ree 1997). Pollen size was similar between species, but the observed pollen dimorphism was insufficient to distinguish between morphs. Consequently, it was not possible to accurately determine the rate of legitimate pollination (Ornelas *et al.* 2004; Valois-Cuesta *et al.* 2011; Furtado *et al.* 2021, 2023), the extent of pollen transfer between species in congeneric patches (Wesselingh *et al.* 2000; Valois-Cuesta *et al.* 2011), or the interaction between these factors (Pérez-Barrales and Armbruster 2023). The fact that we observed bumblebees moving between species, and the similar reproductive organ architecture suggests that pollen transfer probably occurred between *Palicourea* species. In contrast, the observation of heterospecific pollen transfer from non-congeneric coflowering species was anecdotal. We acknowledge that precise measurements of legitimate pollination or distinction of pollen between the two *Palicourea* species would have provided more accurate estimates to measure the function of pollination and reproductive interference between the species, which remains an important area for future research.

Despite the limitations described above, the observed pollen deposition patterns offer valuable insights into pollinator visitation. It can be hypothesized that *P. coriacea* flowers surveyed during the early period and in congeneric patches with *P. officinalis* during the coflowering period should experience similar pollen deposition values since these conditions had lower bumblebee visitation patterns. Furthermore, the higher bumblebee visitation observed in the coflowering period in conspecific *P. coriacea* patches should correspond with larger pollen deposition values (Jennersten *et al.* 1988; Galen 1992; Engel and Irwin 2003). However, stigmas in congeneric patches generally exhibited either similar pollen loads (for T flowers) or more pollen (for P flowers) compared to flowers from the early period or those in conspecific patches. This suggests that fewer visits may be required to adequately cover the stigmatic surface with pollen, implying that additional visits do not necessarily translate into increased pollen deposition. An alternative explanation could involve the length range and spatial distribution of stamens, the placement of pollen along the bodies of bumblebees, and the sexual architecture hypothesis (Murcia and Feinsinger 1996). Following the concept of fundamental inaccuracy and the mechanical fit between flowers and pollinators (Armbruster *et al.* 2009a, b), it is expected that bumblebees visiting patches with only one species (i.e. during the early period and in conspecific patches) should have pollen placed according to the contact area of P and T anthers. This area is represented by the average height of those organs (9.98 mm to 11.00 mm, respectively, Table 3). In contrast, bumblebees visiting patches with two species would have greater body surface area covered with pollen due to the variation in average anther height in conspecific patches, ranging from 9.98 mm (Pin anthers of *P. coriacea*) to 14.20 mm (Thrum anthers of *P. officinalis*, Table 3). Thus, the likelihood of stigma contact with a pollen-covered body part might be higher in conspecific patches. How reproductive organ variation affects pollen flow between species deserves attention to quantify reproductive interference, particularly given the similarity in organ height between species.

## Concluding remarks

The primary objective of this study was to assess whether the efficiency in the pollen delivery and deposition on the stigmas of *P. coriacea* declines when it coflowers with *P. officinalis*, and to explore potential axes of pollination niche differentiation. Our findings suggest that sequential flowering could be important in minimizing pollinator sharing and reducing pollen transfer between these species (Botes *et al.* 2008; Souza *et al.* 2017). Flowering phenology is a phylogenetically conserved trait, so it could function to reduce competition for pollination, but not necessarily evolve in response to competition (Kochmer and Handel 1986; Strauss *et al.* 2021). By integrating the variation in flowering between species with pollinator syndromes and preferences, we were able to evaluate pollination function more comprehensively. The observed floral traits, along with previous pollinator data, aligned with the morphological characteristics of bee and hummingbird pollination syndromes, which could represent significant axes of niche differentiation (Fenster *et al.* 2004; Rosas-Guerrero *et al.* 2014; Dellinger 2020). In our study, it remains inconclusive whether the two *Palicourea* species partition the pollinator niche, since we observe *B. pauloensis* visits in the two species, and we lacked observations from *P. officinalis* in conspecific patches and throughout the entire flowering season. Nonetheless, the combined data on visitation rates, pollen delivery, and deposition patterns suggest the occurrence of reproductive interference. Despite an apparent constancy on *P. coriacea* flowers, visits of bumblebees between species in congeneric patches could affect the efficiency of legitimate pollen transfer and promote pollen transfer between species. Taken together, our results beg for future research to further elucidate pollen delivery and deposition dynamics throughout the flowering season.

## Supplementary Material

plaf014_suppl_Supplementary_Tables_S1-S7

## Data Availability

All data can be found in the Zenodo repository with DOI 10.5281/zenodo.14946920.
