# Supplementary material for: Bumblebee visitation and pollen dynamics in Palicourea coriacea (Rubiaceae): does coflowering with congeneric species matter?
Source: AoB Plants. 2025 Mar 13;17(3):plaf014. doi: 10.1093/aobpla/plaf014 (PMC12190807; doi:10.1093/aobpla/plaf014)
Supplement: plaf014_suppl_Supplementary_Tables_S1-S7 [file plaf014_suppl_supplementary_tables_s1-s7.docx]

**Table S1.** Estimate values of the fixed term and random factor derived from the generalised linear mixed effect model on all *Bombus pauloensis* visits observed during the two observation periods using visits observed only in *P. coriacea* (excluding visits on *P. officinalis* in conspecific patches during the coflowering period)

| Dependent variable | Visits observed only in *P. coriaceae* | | | |
| --- | --- | --- | --- | --- |
| Predictors | Estimates | Std. Error | Z value | P |
| (Intercept) | 1.0944 | 0.3232 | 3.385 | 0.0007 |
| Flowering period (coflowering) | 0.9564 | 0.2497 | 3.831 | 0.0001 |
| Random Effects |  |  |  |  |
| Variance | 0.5701 |  |  |  |
| Std. Dev | 0.7551 |  |  |  |
| n | 7 |  |  |  |
| N observations | 347 |  |  |  |

**Table S2.** Estimate values of the fixed term and random factor derived from the generalised linear mixed effect model on *Bombus pauloensis* visits observed during the coflowering period on *Palicourea coriacea* in conspecific patches and congeneric patches with *P. officinalis*.

| Predictors | Estimates | Std. Error | Z value | P |
| --- | --- | --- | --- | --- |
| (Intercept) | 1.8882 | 0.2017 | 9.363 | <0.00001 |
| Patch type (conspecific) | 0.6358 | 0.1931 | 3.292 | 0.0001 |
| Random Effects |  |  |  |  |
| Variance | 0.5701 |  |  |  |
| Std. Dev | 0.7551 |  |  |  |
| n | 3 |  |  |  |
| N observations | 191 |  |  |  |

**Table S3.** Estimate values of the fixed term and random factor derived from the generalised linear mixed effect model on *Bombus paulensis* visits observed during the coflowering period on *Palicourea coriacea* and *P. officinalis* using the data from congeneric patches.

| Predictors | Estimates | Std. Error | Z value | P |
| --- | --- | --- | --- | --- |
| (Intercept) | 1.9575 | 0.3834 | 5.105 | <0.00001 |
| Species visited (Palicourea_officinalis) | -0.8568 | 0.3109 | -2.755 | 0.006 |
| Random Effects |  |  |  |  |
| Variance | 0.3176 |  |  |  |
| Std. Dev | 0.5636 |  |  |  |
| n | 3 |  |  |  |
| N observations | 181 |  |  |  |

**Table S4**. Estimate values of the fixed terms and random factor derived from the generalised linear mixed effect model on pollen delivery (measured as pollen left in anthers) and pollen deposition on the stigmas of *Palicourea coriacea* Pin and Thrum flowers during the two flowering periods of observation (early period and coflowering with *P. officinalis*) of the flowers sampled in the morning and afternoon (sampling time)

| Dependent variable:  Pollen counts in anthers | Pin anthers | | | | Thrum anthers | | | |
| --- | --- | --- | --- | --- | --- | --- | --- | --- |
| Predictors | **Estimates** | **Std.**  **Error** | **Z value** | **P** | **Estimates** | **Std.**  **Error** | **Z value** | **P** |
| (Intercept) | 6.6325 | 0.1165 | 56.95 | <0.00001 | 6.5309 | 0.1266 | 51.60 | <0.00001 |
| Flowering period (coflowering) | -0.1140 | 0.1462 | -0.78 | 0.4354 | -0.3950 | 0.1698 | -2.33 | 0.02 |
| Sampling time (afternoon) | -0.4070 | 0.2013 | -2.02 | 0.04 | -0.3975 | 0.2109 | -1.88 | 0.06 |
| Interaction (coflowering:afternoon) | 0.1353 | 0.2511 | 0.54 | 0.59 | 0.1620 | 0.2818 | 0.57 | 0.57 |
| Random effect |  |  |  |  |  |  |  |  |
| Variance | 0.0296 |  |  |  | 0.138 |  |  |  |
| Std. Dev | 0.1722 |  |  |  | 0.3714 |  |  |  |
| n | 49 |  |  |  | 50 |  |  |  |
| N observations | 148 |  |  |  | 257 |  |  |  |
|  |  |  |  |  |  |  |  |  |
| Dependent variable:  Pollen counts on the stigmas | **Pin stigmas** | | | | **Thrum stigmas** | | | |
| Predictors | **Estimates** | **Std.**  **Error** | **Z value** | **P** | **Estimates** | **Std.**  **Error** | **Z value** | **P** |
| (Intercept) | 4.03722 | 0.17449 | 23.137 | <0.00001 | 4.2860 | 0.1045 | 41.00 | <0.00001 |
| Flowering period (coflowering) | 0.60538 | 0.22153 | 2.733 | 0.00628 | 0.5406 | 0.1394 | 3.88 | 0.00011 |
| Sampling time (afternoon) | 0.16823 | 0.29294 | 0.574 | 0.57 | 0.1681 | 0.1742 | 0.96 | 0.34 |
| Interaction (coflowering:afternoon) | -0.01084 | 0.37019 | -029 | 0.98 | 0.1128 | 0.2302 | 0.49 | 0.62 |
| Random effect |  |  |  |  |  |  |  |  |
| Variance | 0.203 |  |  |  | 0.06682 |  |  |  |
| Std. Dev | 0.4506 |  |  |  | 0.2585 |  |  |  |
| n | 49 |  |  |  | 50 |  |  |  |
| N observations | 146 |  |  |  | 248 |  |  |  |

**Table S5**. Predicted estimate, standard error (s.e.) and sample size (n) of pollen delivery (estimated from pollen counts in anthers) and pollen deposition on the stigmas of Pin and Thrum flowers of *Palicourea coriacea* derived from the GLMMs in Table S4 during the two flowering periods (early and coflowering with *P. officinalis*) and in samples collected in the morning and afternoon (sampling time)

| Dependent variable:  Pollen counts in anthers | | Pin anthers | | | Thrum anthers | |  |
| --- | --- | --- | --- | --- | --- | --- | --- |
| Flowering period | **Sampling time** | Estimate | s.e. | n | Estimate | s.e. |  |
| Early | **Morning** | 759.38 | 88.44 | 34 | 686.03 | 86.82 | 73 |
| Early | **Afternoon** | 505.46 | 82.71 | 17 | 461.01 | 77.78 | 42 |
| Coflowering | **Morning** | 677.57 | 59.15 | 66 | 462.18 | 52.36 | 98 |
| Coflowering | **Afternoon** | 516.33 | 67.43 | 31 | 365.20 | 54.36 | 44 |
|  |  |  |  |  |  |  |  |
| Dependent variable:  Pollen counts on the stigmas | | **Pin stigmas** | |  | **Thrum stigmas** | |  |
| Flowering period | **Sampling time** | Estimate | s.e. | n | Estimate | s.e. | n |
| Early | **Morning** | 56.67 | 9.89 | 34 | 72.67 | 7.60 | 70 |
| Early | **Afternoon** | 67.05 | 15.94 | 17 | 85.98 | 12.141 | 40 |
| Coflowering | **Morning** | 103.81 | 14.29 | 64 | 124.78 | 11.44 | 91 |
| Coflowering | **Afternoon** | 121.51 | 21.89 | 31 | 165.24 | 19.79 | 47 |

**Table S6.** Estimate values of the fixed terms and random factor derived from the generalised linear mixed effect model on pollen delivery (measured as pollen left in anthers) and pollen deposition on the stigmas of *Palicourea coriacea* Pin and Thrum flowers during the coflowering period in flowers sampled in conspecific (only *P. coriacea)* and conspecific patches with *P. officinalis* (patch type) in the morning and afternoon (sampling time).

| Dependent variable:  Pollen counts in anthers | Pin anthers | | | | Thrum anthers | | | |
| --- | --- | --- | --- | --- | --- | --- | --- | --- |
| Predictors | **Estimates** | **Std.**  **Error** | **Z value** | **P** | **Estimates** | **Std.**  **Error** | **Z value** | **P** |
| (Intercept) | 6.6813 | 0.1192 | 56.07 | <0.00001 | 6.3649 | 0.1760 | 16.17 | <0.00001 |
| Patch type (conspecific) | -0.2791 | 0.1571 | -1.78 | 0.08 | -0.4414 | 0.2446 | -1.80 | 0.07 |
| Sampling time (afternoon) | -0.0755 | 0.2064 | -0.37 | 0.71 | -0.1559 | 0.3067 | -0.51 | 0.61 |
| Interaction (conspecific:afternoon) | -0.4009 | 0.2767 | -1.45 | 0.15 | -0.0867 | 0.4087 | -0.21 | 0.83 |
| Random effect |  |  |  |  |  |  |  |  |
| Variance | 5.068e-09 |  |  |  | 0.159 |  |  |  |
| Std. Dev | 7.119e-05 |  |  |  | 0.399 |  |  |  |
| n | 28 |  |  |  | 28 |  |  |  |
| N observations | 97 |  |  |  | 142 |  |  |  |
|  |  |  |  |  |  |  |  |  |
| Dependent variable:  Pollen counts on the stigmas | **Pin stigmas** | | | | **Thrum stigmas** | | | |
| Predictors | **Estimates** | **Std.**  **Error** | **Z value** | **P** | **Estimates** | **Std.**  **Error** | **Z value** | **P** |
| (Intercept) | 4.9334 | 0.1907 | 25.88 | <0.00001 | 4.9580 | 0.1238 | 40.05 | <0.00001 |
| Patch type (conspecific) | -0.5108 | 0.2495 | -2.05 | 0.04 | -0.2619 | 0.1714 | -1.53 | 0.13 |
| Sampling time (afternoon) | 0.1830 | 0.3066 | 0.60 | 0.55 | 0.0983 | 0.2167 | 0.45 | 0.65 |
| Interaction (conspecific:afternoon) | -0.0738 | 0.4092 | -1.18 | 0.85 | 0.34842 | 0.2870 | 1.22 | 0.22 |
| Random effect |  |  |  |  |  |  |  |  |
| Variance | 0.1481 |  |  |  | 0.05178 |  |  |  |
| Std. Dev | 0.3848 |  |  |  | 0.2276 |  |  |  |
| n | 28 |  |  |  | 28 |  |  |  |
| N observations | 95 |  |  |  | 138 |  |  |  |

**Table S7.** Predicted estimate and s.e. of pollen delivery (estimated from pollen counts in anthers) and pollen deposition on the stigmas of Pin and Thrum flowers of *Palicourea coriacea* derived from the GLMMs in Table S6 using the samples collected in the coflowering period in conspecific patches of *P. coriacea* and conspecific patches with P. officinalis (patch type) in samples collected in the morning and afternoon (sampling time)

| Dependent variable:  Pollen counts in anthers | | Pin anthers | | | Thrum anthers | |  |
| --- | --- | --- | --- | --- | --- | --- | --- |
| Patch type | **Sampling time** | Estimate | s.e. | n | Estimate | s.e. | n |
| Conspecific | **Morning** | 603.21 | 61.73 | 38 | 373.72 | 63.53 | 47 |
| Conspecific | **Afternoon** | 374.59 | 57.38 | 17 | 293.20 | 61.91 | 27 |
| Congeneric | **Morning** | 797.36 | 95.01 | 38 | 581.11 | 102.25 | 51 |
| Conspecific | **Afternoon** | 739.36 | 124.60 | 14 | 497.21 | 124.69 | 17 |
|  |  |  |  |  |  |  |  |
| Dependent variable:  Pollen counts on the stigmas | | **Pin stigmas** | |  | **Thrum stigmas** | |  |
| Patch type | **Sampling time** | Estimate | s.e. | n | Estimate | s.e. | n |
| Conspecific | **Morning** | 83.31 | 13.50 | 37 | 109.53 | 13.03 | 45 |
| Conspecific | **Afternoon** | 92.92 | 20.21 | 17 | 171.39 | 25.16 | 28 |
| Congeneric | **Morning** | 138.85 | 26.47 | 27 | 142.32 | 17.6 | 46 |
| Conspecific | **Afternoon** | 166.74 | 40.23 | 14 | 157.02 | 27.83 | 19 |
